# Microbial Load and Diversity of Bacteria in Wild Animal Carcasses Sold as Bushmeat in Ghana

**DOI:** 10.3390/pathogens14080754

**Published:** 2025-07-31

**Authors:** Daniel Oduro, Winnifred Offih-Kyei, Joanita Asirifi Yeboah, Rhoda Yeboah, Caleb Danso-Coffie, Emmanuel Boafo, Vida Yirenkyiwaa Adjei, Isaac Frimpong Aboagye, Gloria Ivy Mensah

**Affiliations:** 1 Department of Animal Biology and Conservation Science, University of Ghana, Legon/Accra P.O. Box LG 67, Ghana; 2 Department of Immunology, Noguchi Memorial Institute for Medical Research, University of Ghana, Legon/Accra P.O. Box LG 581, Ghana; 3 Department of Bacteriology, Noguchi Memorial Institute for Medical Research, University of Ghana, Legon/Accra P.O. Box LG 581, Ghana

**Keywords:** bushmeat, bacteria, microbial load, microbial safety, Ghana, One Health

## Abstract

The demand for wild animal meat, popularly called “bushmeat”, serves as a driving force behind the emergence of infectious diseases, potentially transmitting a variety of pathogenic bacteria to humans through handling and consumption. This study investigated the microbial load and bacterial diversity in bushmeat sourced from a prominent bushmeat market in Kumasi, Ghana. Carcasses of 61 wild animals, including rodents (44), antelopes (14), and African civets (3), were sampled for microbiological analysis. These samples encompassed meat, intestines, and anal and oral swabs. The total aerobic bacteria plate count (TPC), *Enterobacteriaceae* count (EBC), and fungal counts were determined. Bacterial identification was conducted using MALDI-TOF biotyping. Fungal counts were the highest across all animal groups, with African civets having 11.8 ± 0.3 log_10_ CFU/g and 11.9 ± 0.2 log_10_ CFU/g in intestinal and meat samples, respectively. The highest total plate count (TPC) was observed in rodents, both in their intestines (10.9 ± 1.0 log_10_ CFU/g) and meat (10.9 ± 1.9 log_10_ CFU/g). In contrast, antelopes exhibited the lowest counts across all categories, particularly in EBC from intestinal samples (6.1 ± 1.5 log_10_ CFU/g) and meat samples (5.6 ± 1.2 log_10_ CFU/g). A comprehensive analysis yielded 524 bacterial isolates belonging to 20 genera, with *Escherichia coli* (18.1%) and *Klebsiella* spp. (15.5%) representing the most prevalent species. Notably, the detection of substantial microbial contamination in bushmeat underscores the imperative for a holistic One Health approach to enhance product quality and mitigate risks associated with its handling and consumption.

## 1. Introduction

Wild animal meat (bushmeat) is a delicacy in many parts of the world, especially in Africa [1]. Popular bushmeat in Africa includes grasscutters, chimpanzees, and bushbucks. Intestines and their contents, meat, and muscles of wild animals are normally used in food preparation by local communities [1,2]. Bushmeat satisfies diverse nutritional requirements, including reduced cholesterol levels, high protein content, and beneficial fat profiles [3,4]. It is widely sought after for its perceived medicinal and health benefits. Grasscutter meat provides lean, low-cholesterol protein and essential minerals, and its digestion is rapid when properly prepared [3]. Antelope meat is known to exhibit similar leanness and nutrient density, with additional advantages such as omega-3 fatty acids, Conjugated Linoleic Acid (CLA), and antioxidant vitamins or minerals [5]. These characteristics align with reports suggesting wild game as a healthier alternative to conventional meat, particularly for cardiovascular health, immune support, and antioxidant intake [5]. Additionally, bushmeat serves as a substantial source of income for various stakeholders within the value chain, including hunters, market traders, food vendors, and other key participants in the production process [3,6]. As such, the sale of bushmeat plays a vital role in supporting livelihoods and enhancing food security across numerous African countries [1,7]. In Ghana, the bushmeat industry is estimated to generate an annual retail value of $48,000 and accounts for approximately 75% of meat consumption in rural areas [8].

The trade and consumption of bushmeat, while significant in many regions, present notable health risks. Bushmeat has been linked to the transmission of zoonotic and foodborne diseases, exposure to antimicrobial-resistant bacteria, and the potential for disease outbreaks and pandemics. Historical data indicate that the handling and consumption of wild animal meat have contributed to the emergence and spread of various diseases [1]. The global community continues to grapple with the aftermath of the SARS-CoV-2 pandemic, which has been associated with wildlife consumption [4]. Additionally, microbial contamination in meat remains a critical food safety concern [9]. According to a 2025 report from the Centers for Disease Control and Prevention (CDC), major foodborne pathogens are responsible for approximately 1.9 billion cases of illness, leading to 56,000 hospitalizations and 1350 deaths annually in the United States [10]. Similarly, in sub-Saharan Africa, diarrheal diseases account for around 550 million hospitalizations and 230,000 deaths each year [10].

Microbial contamination of bushmeat can occur through diverse pathways, presenting substantial public health risks. Wild animal hunting is commonly conducted under uncontrolled and unhygienic conditions, particularly in sub-Saharan Africa. Furthermore, the transportation of these products to distant markets often does not comply with established sanitary protocols [11].

Additionally, wildlife inhabiting natural ecosystems is increasingly exposed to pathogenic bacteria due to anthropogenic activities. Factors such as improper waste disposal, water pollution, industrial development, and agricultural and aquacultural operations contribute to the dissemination of microbes into the environment via mediums like water and air [12]. This heightened exposure has facilitated the evolution of numerous pathogenic microorganisms, exerting detrimental effects on both animal and human populations.

Bacterial pathogens commonly associated with meat contamination include *Yersinia enterocolitica*, diarrheagenic *Escherichia coli*, *Staphylococcus aureus*, and *Salmonella* sp. [12]. Consequently, these bacteria have been the primary focus of food safety research and policy development [13]. However, emerging evidence indicates that microbial contamination in bushmeat extends beyond these well-known pathogens. Opportunistic bacteria from the *Enterobacteriaceae* family, such as *Enterococcus* spp. and extraintestinal *E. coli*, are increasingly being transmitted from wild animals to humans through the handling and consumption of bushmeat [13]. This study evaluated the microbial load and diversity of bacteria present in four distinct body parts of wild animals, aiming to deliver a comprehensive analysis of bacterial contamination. By identifying areas of elevated risk and comparing contamination levels across different carcass parts, the research sought to uncover critical patterns. The study’s findings provide valuable insights into the presence and diversity of pathogens linked to wild animal meat. These insights are pivotal for conducting quantitative risk assessments related to zoonotic disease emergence, strengthening surveillance of potential spillover events, and guiding targeted food safety and public health interventions.

## 2. Materials and Methods

### 2.1. Approval and Permission

Permission was sought from the Wildlife Division of the Forestry Commission of Ghana, which regulates activities at the bushmeat hub used in this study.

### 2.2. Study Area and Design

**Figure 1:**
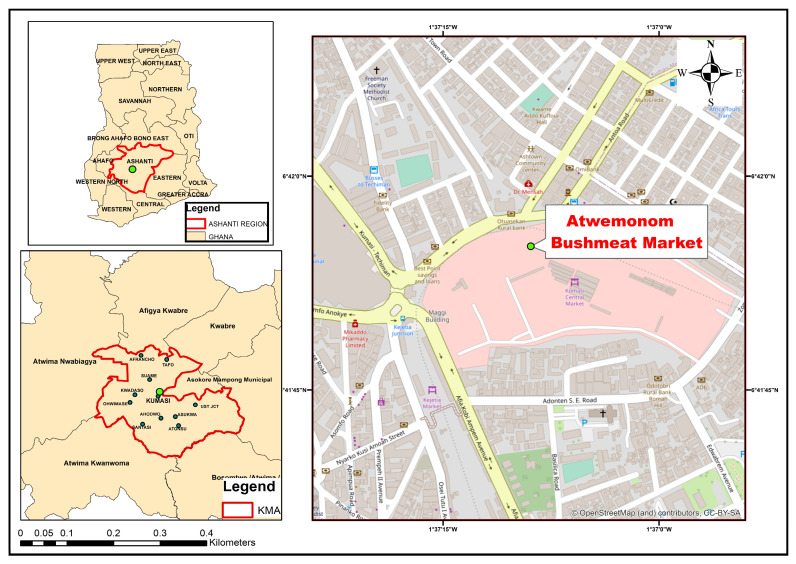
Map showing the location of Atwemonom bushmeat market.

This cross-sectional study utilized samples conveniently obtained from wild animal carcasses being processed for sale at the Atwemonom bushmeat market (6.698635° N, 1.619140° W), which is situated within the central business district of Kumasi, in the Ashanti region of Ghana (https://www.sciencedirect.com/science/article/pii/S2213224424001019, accessed on 21 July 2025) (Figure 1). Atwemonom, literally translating to “the place for freshly killed duikers”, serves as the primary fresh bushmeat market in Southern Ghana, where wild animal carcasses are traded. Hunters from various localities within the southern and middle belts of Ghana retail their carcasses at this market, thereby representing the overall trade in fresh bushmeat in southern Ghana [14]. Fresh carcasses are sold to traders in the early morning as hunters conclude their nocturnal hunts. These carcasses are subsequently processed and sold to both individuals and local city restaurants, commonly referred to as “chop bars” [14].

### 2.3. Sample Collection

Upon arrival at the Atwemonom market in Kumasi, Ghana, hunters, predominantly male, delivered freshly killed and unprocessed wild animal carcasses. These animals, generally hunted the night before using traps, snares, or firearms, were typically transported in sacks or carried over the shoulder.

Before dressing and processing carcasses, trained personnel collected oral and rectal swabs using sterile nylon-flocked swabs (FLOQSwabs^®^, 501CS01; Copan Diagnostics, Murrieta, CA, USA), designed to optimize bacterial and DNA recovery. For oral swabs, the swab was inserted approximately 2 cm beyond the oral cavity, while rectal swabs were collected 2 cm past the anal verge. Each swab was immediately placed into a sterile 15 mL Falcon tube containing Cary–Blair transport medium (Oxoid CM0519B, Basingstoke, UK). Oral and rectal swabs play a critical role in pre-harvest monitoring by identifying animals actively shedding pathogens prior to slaughter, serving as early indicators of potential contamination risks during evisceration and carcass processing [15]. Although these swabs do not directly assess meat contamination, they serve as early indicators of animals that may pose a contamination risk during evisceration and carcass processing [14].

Following swab collections, carcasses were singed over open flames for 5–10 min, with the duration adjusted based on the animal’s size. Typically, hardwood logs were arranged directly on the ground, using minimal equipment like a brazier. This singeing process effectively removed fur and surface contaminants, preparing the carcass for further processing. Once singed, the carcasses were thoroughly washed with flowing water to ensure cleanliness. They were then transferred to designated dressing tables for evisceration and meat sectioning. A sterilized knife was carefully used to open the abdominal cavity, and the intestines were gently extracted to minimize the risk of fecal contamination. Approximately 10 g of intestines containing mostly digested food remnants were collected into sterile Ziplock bags following aseptic incision in the ileum with sterile scissors. A previous study conducted in Ghana revealed that over 40% of Ghanaian consumers of bushmeat exhibited a preference for the intestines and intestinal contents [2]. Additionally, a sterile bush knife was employed to excise 10 g of raw meat from the muscle-rich hindlimb. This procedure was meticulously followed, including careful weighing and placement of the excised meat in labeled Ziplock bags without the skin to minimize external contamination. The samples were subsequently weighed on a CAS-LP 10,000N meat scale (CAS Corporation, Seoul, Republic of Korea) and stored in designated cold boxes.

In total, 61 wild animals were sampled, comprising 44 grasscutters (*Thryonomys swinderianus*), 14 antelopes, and 3 African civets. From each animal, four samples were collected: oral swab, rectal swab, intestines, and meat (Table 1).

The swab samples were securely stored at 4 °C, while intestinal and meat samples were preserved at −20 °C at the Kwame Nkrumah University of Science and Technology (KNUST) throughout a 3-day field study. Following this period, all samples were transported under strict cold chain conditions to the Noguchi Memorial Institute for Medical Research in Accra for comprehensive microbiological analysis.

### 2.4. Microbiological Analysis

#### 2.4.1. Enumeration of Total Aerobic Bacteria, *Enterobacteriaceae*, and Fungi in Wild Animal Carcasses and Intestinal Content

The procedure, described by Adjei et al. [15], was used. Ten (10) grams each of meat and intestinal samples were aseptically homogenized in 90 mL phosphate-buffered saline (PBS) (Thermo Fisher Scientific, Waltham, MA, USA). One (1) ml of the homogenate was diluted in 9 mL PBS to obtain a 10^1^ dilution. Serial dilutions up to 10^−9^ were prepared for the colony count. An aliquot of 1 mL of each dilution was transferred to three Petri dishes (4-inch diameter) labelled plate count agar (PCA), Violet Red Bile Agar (VRBG), and Sabouraud Dextrose Agar (SDA). Molten plate count agar (PCA) (Oxoid CM0325, Basingstoke, UK), Violet Red Bile Agar (VRBG) (Oxoid CM1082, Basingstoke, UK), and Sabouraud Dextrose Agar (SDA) (Oxoid CM0041, Basingstoke, UK) were poured into the plates, respectively. Plates were gently swirled to uniformly mix the inoculum with the molten agar and left to solidify at room temperature. The PCA and VRBG plates were then inverted and incubated at 37 °C for 24 h, while the SDA plates were incubated for 48 h at room temperature.

Following incubation, all Petri dishes were examined, and those with discrete bacterial colonies ranging from 30 to 300 were selected for calculation of colony-forming units (CFU). Pale yellow colonies on PCA, pink colonies on VRBG, and creamy white colonies on SDA were counted using a digital colony counter (Rocker Scientific, Taiwan, Galaxy 330, 175330-01) for estimation of total plate, *Enterobacteriaceae*, and fungal count, respectively.

Petri dishes with colony counts below 30 or above 300 were considered too few to count (TFTC) or too numerous to count (TNTC), respectively, and were thus excluded from the analysis. Enumeration of colonies was performed using the CFU formula from the UNI EN ISO 7218:2013 reference standard, as shown in the following formula [16]:

CFU/g = (number of colonies × dilution factor)/volume of culture plated

The results were reported as log_10_ CFU/g. Total plate count (TPC), *Enterobacteriaceae* count (EBC), and fungal counts exceeding log_10_ 7 CFU/g were considered unsafe for human consumption and handling, as indicated by the International Commission on Microbiological Specifications for Foods (ICMSF) [17]. According to the Ghana Standards Authority (GSA), the acceptable TPC for raw meat or food should not exceed 10^6^ CFU/g (log_10_ 6 CFU/g) [15].

#### 2.4.2. Culture and Identification of Bacteria

For meat samples, a loopful of the homogenate prepared during serial dilution was used as the inoculum. Anal and oral swabs, as well as the intestinal content of each animal carcass that was originally kept in Cary–Blair agar, were cultured directly on various agar for bacteria isolation. Samples were cultured on MacConkey agar (Oxoid CM007, Basingstoke, UK) to recover gram-negative bacteria, as well as on Bacillus agar (Oxoid CM0617, Basingstoke, UK), Campylobacter agar (Oxoid CM0739, Basingstoke, UK), and Chromogenic UTI agar (Oxoid CM1050, Basingstoke, UK). Agar plates were freshly prepared a day before culturing. Cultures were performed under sterile conditions in a biosafety cabinet. All plates were incubated at 37 °C for 24 h and then examined for growth. Isolated bacterial colonies were selected and sub-cultured based on colony characteristics. Three single bacterial colonies were selected from sub-cultured plates and sub-cultured on individual nutrient agar (Oxoid CM0004, Basingstoke, UK) plates for 24 h to obtain pure colonies for identification.

#### 2.4.3. Identification of Bacteria Species Using MALDITOF Mass Spectrometry

Bacteria isolates from nutrient agar plates were identified using a mass spectrometer as described by Alcalá, et al. [18]. Briefly, 1 μL of bacteria from a pure colony on the nutrient agar plate was spotted on the MALDI-TOF target plate using a sterile loop. A protein extraction step was carried out by adding 1 μL of 100% formic acid (Sigma-Aldrich, St. Louis, MO, USA), which was allowed to dry at room temperature. Once dried, the sample was overlaid with 1 μL of α-HCCA matrix (α-cyano-4-hydroxycinnamic acid) obtained from Thermo Fisher Scientific, Waltham, MA, USA. After the matrix dried, the MALDITOF plate was inserted into a designated portion of the mass spectrometer. Spectra acquisition was performed using the default settings of the mass spectrometer, and the obtained spectra were compared with a database for identification. A bacterial test standard provided by the manufacturer was included in each run to ensure proper calibration of the MALDI-TOF MS instrument. Each bacterial isolate was analyzed in duplicate, and the higher score value from the two runs was recorded. In this study, score values were used to assess the confidence of bacterial identification. A score value of ≥2.0 was established as the threshold for high-confidence identification, indicating a strong match between the acquired spectra and the database. A score value of ≥1.7 was set for low-confidence identification, indicating a weaker match, but still considered a possible identification. These score ranges helped categorize the reliability of the results obtained from the MALDI-TOF analysis. All isolates were analyzed using the Microflex LT benchtop mass spectrometer (Bruker Daltonics, Bremen, Germany).

### 2.5. Data Analysis

Statistical analyses were performed using GraphPad Prism (version 10.2.1). The counts were expressed in log colony-forming units per gram of sample [Log10 colony-forming unit (CFU)/g]. Values for microbial loads were reported as means ± standard error (SE). A one-way analysis of variance test was conducted together with an LSD. Bacterial species frequency was calculated by dividing the number of specific bacteria isolates by the total number of bacteria isolated, and the ratio was expressed as a percentage. The Kruskal–Wallis test was used to compare the differences in bacterial prevalence among the three animal groups: rodents, antelopes, and African civets. Dunn’s multiple comparison test was then used to determine the specific differences between the groups.

## 3. Result

### 3.1. Microbial Load of Meat and Intestines of Wild Animal Carcasses

We determined the microbial load in the meat and intestines of the three animal groups. Across all species, intestinal samples consistently harbored higher microbial counts than meat, emphasizing the gastrointestinal tract as a primary reservoir of microbial contamination.

These findings underscore the potential public health risks associated with bushmeat handling and consumption, particularly when sanitary practices are lacking. Improved hygiene during field dressing and carcass processing, combined with increased public awareness and routine surveillance, is essential for mitigating foodborne and zoonotic disease transmission.

#### 3.1.1. Microbial Load of Intestines of Wild Animal Carcasses (Bushmeat)

**Figure 2:**
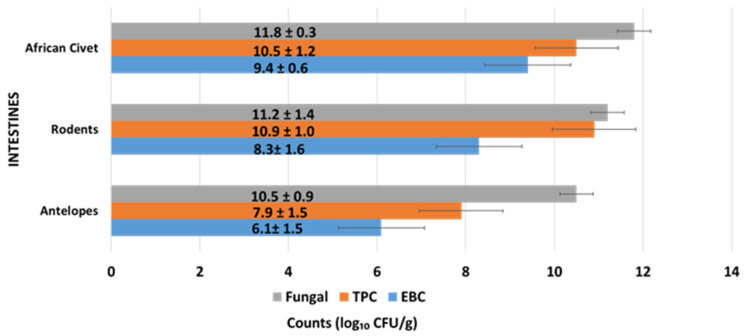
Microbial load (mean log_10_ CFU/g ± SD) in intestinal samples from three wildlife species. Microbial counts are presented as mean log_10_ CFU/g ± standard deviation (SD). TPC = total plate count; EBC = *Enterobacteriaceae* count. Data reflect microbial loads in meat samples from African civets, rodents, and antelopes. Variability in counts suggests differing levels of hygiene, species-specific flora, and post-harvest handling conditions.

Intestinal samples from all three wild animal groups had high microbial loads, as shown in Figure 2. African civets had the highest fungal load (11.8 ± 0.3 log_10_ CFU/g) and TPC (10.5 ± 1.2 log_10_ CFU/g), along with relatively high *Enterobacteriaceae* counts (9.4 ± 0.6 log_10_ CFU/g). Rodents followed closely with slightly lower fungal (11.2 ± 1.4 log_10_ CFU/g) and TPC (8.3 ± 1.6 log_10_ CFU/g) levels, while antelopes had the lowest counts in all categories, especially in EBC (6.1 ± 1.5 log_10_ CFU/g). A significant difference in *Enterobacteriaceae* counts was observed between African civets (9.4 ± 0.6 log_10_ CFU/g) and antelopes (6.1 ± 1.5 log_10_ CFU/g) (*p* < 0.01). These results suggest substantial microbial colonization in the gastrointestinal tracts of wild animals. The lower EBC in antelopes may be due to differences in diet, habitat, or gut microbiota composition.

Microbial counts are expressed as mean log_10_ colony-forming units per gram (CFU/g) ± standard deviation (SD). TPC = total plate count; EBC = *Enterobacteriaceae* count. Values were derived from intestinal samples collected from freshly slaughtered wild animal carcasses under sterile conditions. Differences in microbial loads reflect species-specific gut microbiota and possible exposure to environmental contaminants.

#### 3.1.2. Microbial Load of Meat of Wild Animal Carcasses (Bushmeat)

**Figure 3:**
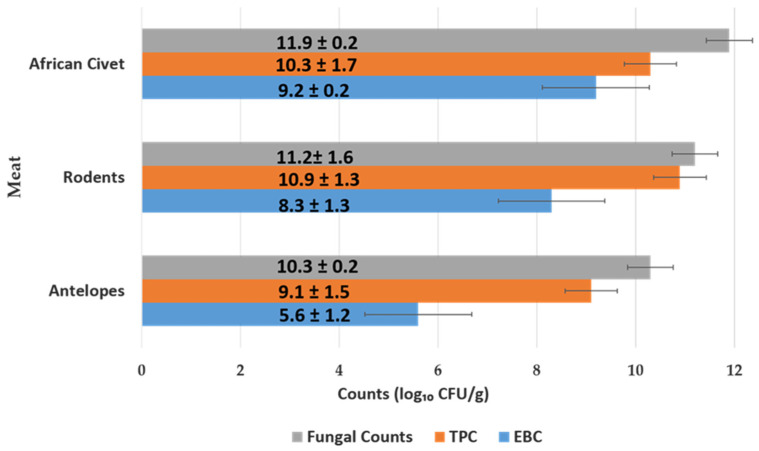
Microbial load (mean log_10_ CFU/g ± SD) in meat samples from three wildlife species.

As illustrated in Figure 3, African civet meat samples had the highest microbial load, with fungal counts averaging 11.9 ± 0.2 log_10_ CFU/g, TPC at 10.3 ± 1.7 log_10_ CFU/g, and EBC at 9.2 ± 0.2. log_10_ CFU/g. Rodents followed closely, with fungal loads of 11.2 ± 1.6 log_10_ CFU/g, TPC of 10.9 ± 1.3 log_10_ CFU/g, and EBC of 8.3 ± 1.3 log_10_ CFU/g. *Enterobacteriaceae* counts in antelope meat (5.6 ± 1.2 log_10_ CFU/g) were significantly lower than those in African civets (9.2 ± 0.2 log_10_ CFU/g) and rodents (8.3 ± 1.3 log_10_ CFU/g), with statistically significant differences observed (0.001 < *p* < 0.01). Antelope meat samples had the lowest microbial contamination across all categories, particularly *Enterobacteriaceae* counts (EBC), with a mean value of 5.6 ± 1.2 log_10_ CFU/g. In contrast, meat from African civets and rodents harbored significantly higher EBC levels, indicating greater enteric bacterial contamination. These differences may be attributed to species-specific gut microbiota, environmental exposure, and variations in handling and processing hygiene.

### 3.2. Prevalence and Diversity of Bacteria Isolated from Wild Animal Carcasses

A total of 524 bacterial isolates, encompassing 20 genera (Table 2), were recovered from 61 wild animal carcasses representing three distinct animal groups. *Escherichia coli* (95 isolates, 18.1%) and *Klebsiella* spp. (81 isolates, 15.5%) emerged as the predominant species, reflecting extensive fecal and environmental contamination.

Rodent samples had the highest bacterial diversity, with 16 distinct species identified. Among the four sample types analyzed, anal swabs accounted for the largest proportion of isolates (152/524, 29.0%), while oral swabs yielded the fewest (117/524, 22.3%). This distribution underscores the gastrointestinal tract as a significant reservoir for bacterial pathogens.

Notably, *E. coli*, *Klebsiella*, *Serratia*, *Pseudomonas*, and *Enterobacter* spp. were consistently isolated across all three animal groups, indicating a broad host range and elevating concerns regarding potential zoonotic transmission. The presence of opportunistic and pathogenic species such as *Yersinia ruckeri*, *Aeromonas* spp., and *Bacillus cereus* highlights the microbial hazards associated with bushmeat handling and consumption.

The pronounced prevalence of enteric bacteria, particularly in anal and intestinal samples, emphasizes the critical need for rigorous hygiene practices during slaughter and processing. These findings underscore the necessity of robust meat inspection protocols, continuous microbial surveillance, and comprehensive public health education to mitigate the risks of foodborne and zoonotic disease transmission linked to bushmeat.

Values represent the number of bacterial isolates recovered from each sample type per animal group. Percentages in parentheses indicate the relative abundance of each bacterial species out of the total 524 isolates. Samples were collected from four anatomical sites: anal swabs, oral swabs, intestinal content, and muscle tissue (meat). Bacterial identification was performed using MALDI-TOF mass spectrometry (Bruker Daltonics, Bremen, Germany). The highest isolate count was observed in anal samples, while oral samples yielded the fewest isolates.

### 3.3. Bacteria Prevalence in Animal Groups

Table 3 presents the prevalence (with confidence intervals) of bacterial species across the respective animal groups. Overall, rodents exhibited the highest bacterial prevalence and diversity, followed by antelopes, with African civets showing the lowest. This pattern was consistent across most bacterial species, with narrower confidence intervals in rodents indicating higher precision due to a larger sample size.

*Escherichia coli* was the most prevalent species, found in all three animal groups. *Serratia* spp. were also commonly isolated, although *Serratia* was notably higher in antelopes and civets. *Proteus mirabilis* showed exclusively high prevalence in civets (100%), despite being rare in other groups. Several opportunistic and potentially pathogenic bacteria, including *Yersinia ruckeri*, *Pseudomonas* spp., and *Enterobacter* spp., were detected across species, indicating a broad host range and zoonotic potential. The reported *p*-value (*p* = 0.03) reflects a statistically significant difference in the overall prevalence of bacterial species among the three animal groups (rodents, antelopes, and African civets).

These findings suggest that rodents may serve as major reservoirs of bacterial pathogens in bushmeat, while the lower precision in estimates for African civets highlights the need for broader sampling. The widespread presence of enteric and opportunistic bacteria reinforces concerns about microbial risks in bushmeat handling and consumption. This underscores the need for improved hygiene, meat inspection, and public health education to mitigate zoonotic disease transmission.

Prevalence values represent the proportion (%) of animals within each group that tested positive for the indicated bacterial species, based on combined results from anal, oral, intestinal, and flesh samples. Confidence intervals (95% CI) indicate the precision of prevalence estimates, calculated using the Wilson method. Significant differences in prevalence were observed between rodents and African civets (A and C, *p* = 0.02) and between antelopes and African civets (B and C, *p* = 0.02), while no significant difference was found between rodents and antelopes (A and B, *p* > 0.05) in all samples.

## 4. Discussion

This study identified distinct patterns of microbial contamination across various anatomical parts and species of wild animal carcasses. We conducted a comprehensive quantification of microbial loads, measuring total plate count (TPC), *Enterobacteriaceae* count (EBC), and fungal counts in both the meat and intestinal samples of African civets, rodents, and antelopes—wild animals commonly consumed as bushmeat. Notably, the intestines, valued as edible organs alongside their semi-digested contents, particularly from herbivores like grasscutters, hold significant culinary importance. These intestines, favored by many Ghanaian bushmeat consumers, are frequently utilized to enhance the flavor of traditional soups and stews [2]. Fungal counts consistently exhibited the highest levels across all sample types, exceeding both total plate counts (TPCs) and *Enterobacteriaceae* counts (EBCs). These findings indicate that fungal contamination represents a predominant microbiological concern in bushmeat derived from these species.

Species-specific differences in microbial loads can be attributed to variations in dietary habits, gut physiology, and environmental exposure. African civets and rodents, which are omnivorous scavengers, often consume decomposing organic matter, increasing their exposure to fungal spores and spoilage organisms. In contrast, antelopes are strict herbivores, feeding primarily on fresh vegetation, which may reduce their contact with fungal and enteric pathogens from decaying materials. Consequently, antelopes exhibited the lowest microbial loads in both meat and intestinal samples.

The gastrointestinal anatomy of these species provides additional insight into the observed differences. Specifically, rodents and civets feature relatively simple digestive tracts, which support the survival of diverse microbial communities, including fungi [19]. Antelopes, as foregut fermenters, maintain a more regulated microbial environment [19] that may inhibit fungal overgrowth. These biological differences likely contribute to the lower fungal counts recorded in herbivores.

Environmental conditions have a profound impact on fungal proliferation, particularly in tropical regions. Ghana’s tropical climate, characterized by consistently high humidity levels ranging from 77% to 85% and average daily temperatures between 24 °C and 30 °C, creates an optimal environment for fungal growth on meat surfaces. Corroborating this, a study conducted at Rumokoro Market in Nigeria reported a higher prevalence of fungal species in rodents—*Aspergillus* at 57.1% and *Penicillium* at 42.9%—compared to antelopes, which exhibited *Aspergillus* at 53.6% and *Penicillium* at 44.4% [7]. Similarly, Benfoh [20] reported high levels of *Aspergillus* and *Penicillium* in dried bushmeat in Ghana, which aligns with our findings of fungal dominance. In contrast, studies involving poultry and pigs raised under hygienic, controlled environments report lower fungal loads compared to those observed in bushmeat and informal market settings [21], underscoring the influence of both environment and animal ecology on microbial contamination in bushmeat.

Fungal counts in this study reached levels exceeding 7.5 log_10_ CFU/g in some samples, significantly surpassing those of other microbial types. Our findings report higher microbial loads than those observed by Ahouanse, Issa-Zacharia [22] in Tanzania, who documented fungal counts of 4.03 ± 0.54 log_10_ CFU/g in antelopes and 3.85 ± 0.47 log_10_ CFU/g in grasscutters. This contrast may reflect not only environmental differences, but also variations in post-slaughter handling and drying techniques between the two regions. Elevated fungal loads increase spoilage risk and exposure to mycotoxins such as aflatoxins, which may lead to opportunistic infections in immunocompromised individuals, gastrointestinal disturbances, or even hepatocellular carcinoma [21]. However, it is important to note that not all fungi produce mycotoxins; for example, many species of *Penicillium* and *Aspergillus* are non-toxigenic.

The total plate count (TPC) serves as a comprehensive indicator of microbial quality, quantifying the number of viable aerobic microorganisms, including bacteria, yeasts, and molds. It is widely utilized to evaluate hygiene practices, processing standards, and overall food safety, encompassing both pathogenic and non-pathogenic microbes.

The elevated TPC and *Enterobacteriaceae* count (EBC) observed in this study indicate suboptimal hygiene practices during carcass handling, dressing, and transportation. A significant contributing factor is fecal contamination, often arising from improper evisceration techniques and the utilization of unclean tools. Key observations include the recurrent use of knives without proper sanitation, the absence of gloves or protective clothing, and inadequate hand hygiene among meat handlers. These hygiene deficiencies may account for the unusually high TPC recorded in antelope meat (9.1 ± 1.5 log_10_ CFU/g), which notably surpassed the microbial load found in their intestinal samples (7.9 ± 1.5 log_10_ CFU/g). This finding contrasts with the general trend observed in the study, where intestinal samples typically exhibited higher microbial counts.

These findings are consistent with prior research conducted in Nigeria and South Africa, which documented comparable TPC levels: 7.6 log_10_ CFU/g in duikers, 8.09 ± 0.15 log_10_ CFU/g in rodents, and 7.92 ± 0.19 log_10_ CFU/g in antelopes [6,7,9]. Amponsah, Ankar-Brewoo [8] reported higher bacterial loads in omnivorous species such as rats and monkeys compared to herbivores like rabbits, underscoring the influence of dietary habits and post-harvest handling practices on microbial contamination. Additionally, Gwladys, Abdulsudi [22] identified elevated TPCs and significant *Enterobacteriaceae* contamination across various bushmeat species, attributing these findings to inadequate hygiene practices within the bushmeat value chain. In contrast, a study conducted in Ghana’s Northern Region revealed lower TPC values in fresh beef (4.75 log_10_ CFU/g), which may be attributed to improved sanitary conditions and reduced market congestion compared to the densely populated Ashanti meat market examined in this study [23].

It is noteworthy that while numerous bushmeat studies have investigated microbial diversity through high-throughput sequencing methodologies, such as 16S rRNA analysis, there remains a deficiency in quantitative data concerning microbial loads (e.g., TPC expressed in CFU/g) [8,24]. This highlights the critical need for standardized microbial quantification protocols in future surveillance initiatives to enhance the accuracy and comparability of results.

Elevated *Enterobacteriaceae* count (EBC) signify fecal contamination, often attributable to inadequate hygiene during slaughter and handling. The method of capture also plays a crucial role in influencing EBC levels. For example, Bonardi, Tansini [25] observed that wild boar carcasses with abdominal gunshot wounds exhibited higher EBC (2.79 log_10_ CFU/cm^2^) due to gastrointestinal perforation compared to those shot in the thoracic region (2.49 log_10_ CFU/cm^2^), underscoring the risk of gut content spillage during field dressing.

Similarly, in Cameroon, over 75% of tools and surfaces used in bushmeat processing were contaminated with *Enterobacteriaceae*, indicating widespread environmental contamination [26]. Elevated EBC values are associated with foodborne illnesses such as diarrhea, typhoid fever, and dysentery, and they also raise concerns about the spread of antimicrobial-resistant strains, including ESBL-producing *Escherichia coli*.

A 2019 metagenomic study from Tanzania identified diverse microbial communities in bushmeat, dominated by *Firmicutes* (67.8%), *Proteobacteria* (18.4%), and minor phyla such as *Cyanobacteria* [24]. This profile is consistent with our recovery of diverse bacterial genera, including *Escherichia*, *Klebsiella*, *Enterobacter*, and *Citrobacter*. Likewise, in Namibia, *E. coli* was detected in 74.6% of fresh game meat samples [27], a prevalence comparable to the rates observed in our rodent and antelope samples.

In Accra, Ghana, studies on ready-to-eat meat products also revealed contamination by enteric pathogens, including *Enterobacter* spp., *Citrobacter*, and *Klebsiella* species [28], reinforcing the ongoing food safety risks in urban markets. Dela et al. [28] further reported high TPCs, along with frequent detection of *E. coli*, *Salmonella*, and *Staphylococcus* species, including multidrug-resistant strains.

The absence of *Salmonella* in our samples is consistent with findings from Amponsah, Ankar-Brewoo [8], who similarly reported no *Salmonella* in smoked bushmeat, and Bonardi, Tansini [25], who found low *Salmonella* prevalence in muscle tissue. However, this may reflect differences in enrichment or isolation protocols, such as pre-enrichment duration, low target organism load, or microbial competition on selective media.

Anal swabs yielded more bacterial isolates than oral swabs, which is consistent with the dense microbial populations found in the distal gut. The oral cavity, by contrast, harbors fewer bacteria due to the presence of antimicrobial enzymes like lysozyme and lower nutrient availability [29].

MALDI-TOF MS was instrumental in swiftly and accurately identifying bacterial isolates from bushmeat samples. A 2024 review underscores the method’s reliability, highlighting its ability to reduce identification time by approximately 24 h compared to conventional biochemical systems, while maintaining high accuracy and cost-efficiency [30]. In food microbiology, MALDI-TOF MS has demonstrated strong performance in identifying a broad range of bacterial organisms from meat samples in contrast to traditional sampling methods; a 2021 study on vacuum-packed lamb meat reported 86–100% concordance at the genus level with partial 16S rRNA sequencing [31]. The efficiency of MALDI-TOF MS was a significant factor in the high level of precision and confidence generated during the analysis of results. These findings confirm that MALDI-TOF MS offers a high-throughput, precise method and is ideal for wildlife meat microbiology and zoonotic pathogen surveillance.

## 5. Conclusions

This study highlights significant microbial contamination in bushmeat from the Atwemonom bush meat market in Ghana, with consistently high levels of fungal counts, total plate count (TPC), and *Enterobacteriaceae* count (EBC) detected across various wildlife species and anatomical parts. Fungal contamination was most prevalent, particularly in omnivorous species like rodents and African civets, while herbivorous antelopes exhibited lower microbial loads.

Elevated TPC and EBC levels may indicate lapses in hygiene during carcass handling, processing, and transport. Higher microbial loads in meat compared to intestinal samples suggest cross-contamination during butchering and inadequate sanitation in high-traffic market environments.

The effective use of MALDI-TOF MS for rapid microbial identification underscores its value in food safety monitoring and zoonotic disease surveillance.

This study underscores the critical need for enhanced hygiene protocols, targeted microbial surveillance, and comprehensive capacity-building initiatives for meat handlers engaged in the bushmeat trade. By providing essential baseline data on microbial risks associated with bushmeat from informal markets, the research highlights the need for integrated public health strategies. Guided by the One Health approach, these strategies aim to improve wildlife handling practices, ensure food hygiene, and safeguard consumer health, thereby promoting a holistic framework for public health and safety.

## 6. Limitations of the Study

This study was conducted in a single major bushmeat market, which, although recognized as a principal hub for bushmeat trade in the country, may not fully represent the diversity of practices, species traded, or consumer behaviors present in other bushmeat markets across Ghana. Consequently, the findings may not be generalizable to all bushmeat hubs within the country, as variations could exist due to regional differences in ecosystem biodiversity, cultural practices, and market dynamics.

Additionally, the study was confined to the wet season, which may have influenced the availability of certain species and market activities. Seasonal variations, such as differences in hunting practices, species abundance, and consumer demand, are critical factors that could affect the outcomes. Therefore, conducting similar research during the dry season would provide valuable comparative data, offering insights into seasonal trends and enhancing the robustness of the study’s conclusions.

## Figures and Tables

**Table 1:** Sample distribution and types collected from wild animal carcasses (bushmeat).

Animal Group	N	Animal Sub-Group	N	Anal Swabs	Oral Swabs	Intestines	Meat
Rodents	44	Grasscutters (*Thryonomys swinderianus*)	44	43	44	42	42
Antelopes	14	Bushbuck (*Tragelaphus scriptus*)	7	6	7	6	6
		Maxwell’s Duiker (*Philantomba maxwellii*)	2	2	2	2	2
		Red flank Duiker (*Cephalophus rufilatus*)	5	5	5	4	4
African Civets	3	African Civet (*Civettictis civetta*)	3	3	3	3	3
Total Number	61		61	59	61	57	55

Data represent the number of individual wild animal carcasses sampled, and the corresponding number of biological samples (anal swabs, oral swabs, intestines, and meat) collected per animal subgroup. Differences in sample counts reflect occasional unavailability or damage to specific tissues at the time of collection.

**Table 2:** Bacteria species isolated from different animal groups.

Bacteria Species	Rodents (*n* = 44)				Antelopes (*n* = 14)				African Civet *(n* = 3)				Total
	Anal	Oral	Intestinal	Meat	Anal	Oral	Intestinal	Meat	Anal	Oral	Intestinal	Meat	
*Escherichia coli*	13	6	18	27	8	5	6	7	3	0	0	2	95 (18.1%)
*Klebsiella* sp.	22	10	22	20	0	5	0	0	0	0	0	2	81 (15.5%)
*Hafnia alevi*	9	20	4	1	6	5	7	0	0	0	0	0	52 (9.9%)
*Serratia* sp.	0	0	0	0	7	0	19	13	0	2	0	0	41 (7.8%)
*Aeromonas* sp.	15	14	0	3	0	5	0	0	0	0	0	0	37 (7.1%)
*Yersinia ruckeri*	21	11	0	0	0	0	0	0	0	0	0	0	32 (6.1%)
*Pseudomonas* sp.	9	4	4	3	0	6	2	0	1	0	3	0	32 (6.1%)
*Enterococcus* sp.	11	1	5	10	4	0	0	0	0	0	0	0	31 (5.9%)
*Enterobacter* sp.	0	4	9	1	0	2	0	8	2	0	0	0	26 (4.9%)
*Macrococcus caseolyticus*	0	5	0	7	5	3	0	5	0	0	0	0	25 (4.8%)
*Moelleralla wisconsensis*	3	2	0	8	0	2	0	0	0	0	0	0	15 (2.9%)
*Acinetobacter* sp.	1	0	11	0	0	0	0	2	0	0	0	0	14 (2.7%)
*Ralstonia mannitolityca*	0	2	0	0	5	0	4	1	0	0	0	0	12 (2.3)
*Staphylococcus* sp.	0	0	5	3	1	0	0	2	0	0	0	0	11 (2.1%)
*Proteus mirabilis*	0	0	2	4	1	0	0	0	0	0	0	0	7 (1.3%)
*Pontoea agglomerans*	3	0	0	0	2	0	0	0	0	0	0	0	5 (1.0%)
*Psychrobacter sanguinis*	0	0	0	3	0	0	0	0	0	0	0	0	3 (0.6%)
*Buttiauxella gaviniae*	0	0	0	0	0	2	0	0	0	0	0	0	2 (0.4%)
*Citrobacter* sp.	0	0	0	0	0	1	0	1	0	0	0	0	2 (0.4%)
*Bacillus cereus*	0	0	0	0	0	0	0	1	0	0	0	0	1 (0.2%)
Total	107	79	80	90	39	36	38	40	6	2	3	4	524 (100%)

**Table 3:** Bacteria prevalence in the three animal groups.

Bacteria Species	A. Rodents *(n* = 44)		B. Antelope (*n* = 14)		C. African Civet (*n* = 3)		*p*-Value
	Prevalence	95% CI	Prevalence	95% CI	Prevalence	95% CI	
*Escherichia coli*	72.7	58.2–83.7	50	26.8–73.2	66.7	58.3–98.3	0.03
*Klebsiella* sp.	65.9	51.1–78.1	35.7	16.3–61.2	33.3	1.71–88.2	
*Hafnia alevi*	47.7	33.8–62.1	64.3	38.8–83.7	0	0–56.2	
*Yersinia ruckeri*	47.7	33.8–62.1	26.3	11.8–48.8	0	0–56.2	
*Pseudomonas* sp.	43.2	29.7–57.8	42.9	21.4–77.4	66.7	11.8–98.3	
*Aeromonas* sp.	31.8	20.0–46.6	35.7	16.3–61.2	0	0–56.2	
*Enterococcus* sp.	25	14.6–39.4	21.4	7.6–47.6	0	0–56.2	
*Enterobacter* sp.	20.5	11.2–34.5	42.9	21.4–67.4	66.7	11.8–98.2	
*Acinetobacter* sp.	18.5	10.4–30.8	14.3	2.5–39.9	0	0–56.3	
*Macrococcus caseolyticus*	18.2	9.5–32.0	35.7	16.3–61.2	0	0–56.2	
*Serratia* sp.	18.2	9.5–32.0	73.3	48.1–89.1	66.7	11.8–98.2	
*Staphylococcus* sp.	15.9	7.9–29.4	7.1	0.4–31.5	0	0–56.4	
*Moelleralla wisconsensis*	15.9	7.9–29.4	14.3	2.5–39.9	0	0–56.2	
*Ralstonia mannitolityca*	13.6	6.4–26.7	50.0	26.8–73.2	0	0–56.2	
*Proteus mirabilis*	6.8	2.3–18.2	7.1	0.4–31.5	100	43.9–100	
*Psychrobacter sanguinis*	2.3	0.1–11.8	0	0–21.5	0	0–56.2	
*Buttiauxella gaviniae*	0	0–8.0	12.5	2.2–36.0	0	0–56.2	
*Bacillus cereus*	0	0–8.0	7.1	0.4–31.4	0	0–56.2	
*Citrobacter* sp.	0	0–8.0	6.7	0.3–29.8	0	0–56.2	

## Data Availability

Data will be made available upon request.

## References

[1] Kurpiers L.A., Schulte-Herbrüggen B., Ejotre I., Reeder D.M. (2016). Bushmeat and Emerging Infectious Diseases: Lessons from Africa, in Problematic Wildlife.

[2] Wajah A., Emikpe B., Asare D., Asenso T., Essel-Cobbinah D. (2022). Preference for grasscutter offal by some consumers in the Greater Accra and Ashanti regions of Ghana. Sokoto J. Vet. Sci..

[3] Aboagye I.F., Nkansa-Gyamfi N.A., Obimpeh M.A., Ansa-Tuah A.K., Owusu E.H. (2019). Wildlife Species as Potential Sources of Human Exposure to Parasitic Pathogens in Accra, Ghana. West Afr. J. Appl. Ecol..

[4] Delahay R.J., de la Fuente J., Smith G.C., Sharun K., Snary E.L., Girón L.F., Nziza J., Fooks A.R., Brookes S.M., Lean F.Z.X. (2021). Assessing the risks of SARS-CoV-2 in wildlife. One Health Outlook.

[5] Quarshie J.T., Cofie J.K., Dewornu F.S., Quaye O., Aikins A.R. (2023). Risk of Heavy Metal Poisoning From Consuming Grasscutter Digesta in Ghana. Environ. Health Insights.

[6] Emelue, Idaewor J. (2018). Assessment Of Microbial Count Loads Of Bush Meats Sold At Different Markets In Benin City, Edo State, Nigeria. Int. J. Agric. Sci..

[7] Ikeh M., Anele B.C., Ogbodo U.A. (2021). Assessment of Microbiological Quality Associated with Ready-to-Eat Bush Meat Sold at Rumuokoro Market in Rivers State. Asian J. Res. Zool..

[8] Amponsah A.S., Ankar-Brewoo G.M., Lutterodt H.E., Ofosu I.W. (2024). Assessing the microbial diversity and proximate composition of smoked-fermented bushmeat from four different bushmeat samples. BioTechnologia.

[9] Rani Z.T., Mhlongo L.C., Hugo A. (2023). Microbial profiles of meat at different stages of the distribution chain from the abattoir to retail outlets. Int. J. Environ. Res. Public Health.

[10] WHO (2025). WHO Estimates of the Global Burden of Foodborne Diseases: Foodborne Disease Burden Epidemiology Reference Group 2022–2025.

[11] Bachand N., Ravel A., Onanga R., Arsenault J., Gonzalez J.-P. (2012). Public health significance of zoonotic bacterial pathogens from bushmeat sold in urban markets of Gabon, Central Africa. J. Wildl. Dis..

[12] Yesilay G., Dos Santos O.A.L., Hazeem L.J., Backx B.P., Kamel A.H., Bououdina M. (2023). Impact of pathogenic bacterial communities present in wastewater on aquatic organisms: Application of nanomaterials for the removal of these pathogens. Aquat. Toxicol..

[13] Bintsis T. (2017). Foodborne pathogens. AIMS Microbiol..

[14] McNamara J. (2014). The Dynamics of a Bushmeat Hunting System Under Social, Economic and Environmental Change.

[15] Adjei V.Y., Mensah G.I., Kunadu A.P.-H., Tano-Debrah K., Ayi I., Addo K.K. (2022). Microbial Safety of Beef Along Beef Value Chains in the Ashaiman Municipality of Ghana. Front. Vet. Sci..

[16] Nonga C.H., Zacharia I., Mkupasi E., Ngowi H. (2023). Assessment of Bacterial contamination and associated risk factors in pork slaughtered and marketed in urban Tanzania. Tanzan. J. Health Res..

[17] Thatcher F.S., Clark D.S., Silliker J.H., Elliott R.P., Baird-Parker A.C. (1968). Microorganisms in Foods.

[18] Alcalá L., Marín M., Ruiz A., Quiroga L., Zamora-Cintas M., Fernández-Chico M.A., Muñoz P., Rodríguez-Sánchez B. (2021). Identifying anaerobic bacteria using MALDI-TOF mass spectrometry: A four-year experience. Front. Cell. Infect. Microbiol..

[19] Skarżyńska M., Leekitcharoenphon P., Hendriksen R.S., Aarestrup F.M., Wasyl D., Karunasagar I. (2020). A metagenomic glimpse into the gut of wild and domestic animals: Quantification of antimicrobial resistance and more. PLoS ONE.

[20] Benfoh D.A., Kwarteng E., Asante J. (2022). Mycological contamination in dried bushmeat sold in Ghanaian markets. Afr. J. Microbiol. Res..

[21] Roque K., Lim G.-D., Jo J.-H., Shin K.-M., Song E.-S., Gautam R., Kim C.-Y., Lee K., Shin S., Yoo H.-S. (2016). Epizootiological characteristics of viable bacteria and fungi in indoor air from porcine, chicken, or bovine husbandry confinement buildings. J. Vet. Sci..

[22] Ahouanse G.G.A., Issa-Zacharia A., Majaliwa N. (2023). Bushmeat Consumption in Africa: A Microbiological Safety Challenge?. Asian Food Sci. J..

[23] Anachinaba I.A., Adzitey F., Teye G.A. (2015). Assessment of the microbial quality of locally produced meat (beef and pork) in Bolgatanga Municipal of Ghana. Internet J. Food Saf..

[24] Katani R., Schilling M.A., Lyimo B., Tonui T., Cattadori I.M., Eblate E., Martin A., Estes A.B., Buza T., Rentsch D. (2019). Microbial diversity in bushmeat samples recovered from the Serengeti ecosystem in Tanzania. Sci. Rep..

[25] Bonardi S., Tansini C., Cacchioli A., Soliani L., Poli L., Lamperti L., Corradi M., Gilioli S. (2021). Enterobacteriaceae and *Salmonella* contamination of wild boar (*Sus scrofa*) carcasses: Comparison between different sampling strategies. Eur. J. Wildl. Res..

[26] Soto S.M., Castellsagués L., Ballén V., Gabasa Y., Mayor P., Brull G.R., Funk S.M., Fa J.E. (2025). Prevalence of bacterial contamination on wild meat processing and cooking surfaces in rural Cameroon. One Health.

[27] Fey P.D., Wickert R.S., Rupp M.E., Safranek T.J., Hinrichs S.H. (2018). The prevalence of non-O157: H7 Shiga toxin-producing *Escherichia coli* (STEC) in Namibian game meat. Vet. Ital..

[28] Dela H., Egyir B., Behene E., Sulemana H., Tagoe R., Bentil R., Bongo R.N., Bonfoh B., Zinsstag J., Bimi L. (2023). Microbiological quality and antimicrobial resistance of Bacteria species recovered from ready-to-eat food, water samples, and palm swabs of food vendors in Accra, Ghana. Int. J. Food Microbiol..

[29] Nalage D., Sontakke T., Biradar A., Jogdand V., Kale R., Harke S., Kale R., Dixit P. (2023). The impact of environmental toxins on the animal gut microbiome and their potential to contribute to disease. Food Chem. Adv..

[30] Calderaro A., Chezzi C. (2024). MALDI-TOF MS: A reliable tool in the real life of the clinical microbiology laboratory. Microorganisms.

[31] Altakhis M., Pillidge C.J., Osborn A.M., Torley P.J., Kaur M. (2021). Assessment of the potential use of MALDI-TOF MS for the identification of bacteria associated with chilled vacuum-packaged lamb meat. Meat Sci..

